# Effects of elicitors from culture filtrate of *Fusarium solani* CL105 on flavonoid production of *Scutellaria baicalensi*s calli

**DOI:** 10.3389/fpls.2024.1383918

**Published:** 2024-06-04

**Authors:** Xiaoxuan Cui, Xin Zhang, Huigai Sun, Yuguang Zheng, Chunyan Su

**Affiliations:** ^1^ College of Pharmacy, Hebei University of Chinese Medicine, Shijiazhuang, China; ^2^ Traditional Chinese Medicine Processing Technology Innovation Center of Hebei Province, School of Pharmacy, Hebei University of Chinese Medicine, Shijiazhuang, China; ^3^ International Joint Research Center on Resource Utilization and Quality Evaluation of Traditional Chinese Medicine of Hebei Province, School of Pharmacy, Hebei University of Chinese Medicine, Shijiazhuang, China; ^4^ State Key Laboratory for Quality Ensurance and Sustainable Use of Dao-di Herbs, National ResourceCenter for Chinese Materia Medica, China Academy of Chinese Medical Sciences, Beijing, China; ^5^ Department of Pharmaceutical Engineering, Hebei Chemical and Pharmaceutical College, Shijiazhuang, China

**Keywords:** elicitor, endophyte, gene expression, flavonoids, *Scutellaria baicalensis*, transcription factors

## Abstract

**Introduction:**

Endophytic fungi can promote secondary metabolite accumulation in medicinal plants. Previously, we observed that the culture filtrate of *Fusarium solani* CL105 promoted flavonoid production in *Scutellaria baicalensis* calli. However, the active ingredients and mechanisms associated with this secondary metabolite accumulation remain unclear.

**Methods:**

This study evaluates the effects of different elicitors from the culture filtrate of *F. solani* CL105 namely, exopolysaccharide (EPS), exoprotein (EP), and other parts (OP), on the flavonoid production in *S. baicalensis* calli by HPLC. Subsequently, the underlying mechanism of EPS induced flavonoid production in *S. baicalensis* calli was revealed by transcriptomics and RT-PCR.

**Results and discussion:**

The results indicated a significant increase in flavonoid production in *S. baicalensis* calli following treatment with EPS. Baicalin (1.40 fold), wogonoside (1.91 fold), and wogonin (2.76 fold) were most significantly up-regulated compared with the control. Transcriptome analysis further revealed up-regulation of key enzyme genes (*CHS, CHI, FNS*, and *F6H*) involved in flavonoid synthesis after 5 days of EPS treatment. Moreover, the expression of *GA2ox* and *CYP707A*—genes involved in gibberellin acid (GA) and abscisic acid biosynthesis (ABA), respectively—were significantly up-regulated. The expression levels of certain transcription factors, including *MYB3, MYB8*, and *MYB13*, were also significantly higher than in controls. Our results indicated that EPS was a main active elicitor involved in promoting flavonoid production in *S. baicalensis* calli. We postulated that EPS might stimulate the expression of *MYB3, MYB8, MYB13, GA2ox*, and *CYP707A*, leading to markedly upregulated *CHS, CHI, FNS*, and *F6H* expression levels, ultimately promoting flavonoid synthesis. This study provides a novel avenue for large-scale in vitro production of flavonoids in *S. baicalensis*.

## 1 Introduction

*Scutellaria baicalensis* Georgi, a perennial herb belonging to the family Labiatae, is a well-known traditional Chinese medicinal herb grown in northern China. Flavonoids and their glycosides are the major compounds in *S. baicalensis* and are classified into two types, namely free flavonoids and flavonoid glycosides, of which baicalein and wogonin are the most abundant flavonoids, while baicalin and wogonoside are the most abundant flavonoid glycosides (Zhao et al., 2019a). These flavonoids have pharmacological effects, including antioxidative, anti-inflammatory, antitumor, antiviral, antimicrobial, hepatoprotective, and neuroprotective properties (Wang et al., 2018). Baicalin, in particular, has demonstrated potential in the treatment of COVID-19 (Miguel et al., 2023). Furthermore, baicalin is clinically used as a post-marketing drug to treat acute and chronic hepatitis. However, the wild resources of *S. baicalensis* are continuing to gradually decrease, and the plant quality is limited by growth years (Yuan et al., 2010; Bai et al., 2020). Thus, tissue culturing provides a potential strategy to overcome these issues regarding *S. baicalensis* availability.

In *S. baicalensis*, flavonoids are synthesized via two pathways. The initial compound, phenylalanine, is produced through the shikimate pathway, which generates cinnamic acid via phenylalanine ammonialyase (PAL). In the aerial parts of *S. baicalensis*, cinnamic acid is converted to scutellarein by a series of enzymes, including cinnamate 4-hydroxylase (C4H), 4-coumarate CoA ligase (4CL), chalcone synthase (CHS), chalcone isomerase (CHI), flavone synthase (FNS), and flavone 6-hydroxylase (F6H). Alternatively, in the roots, baicalein, wogonin, and their glycosides are synthesized. This pathway involves the conversion of cinnamic acid to cinnamoyl-CoA in the presence of cinnamate-CoA ligase (CLL-7). Cinnamoyl-CoA is then converted to pinocembrin by CHS and CHI. Next, pinocembrin is converted by FNS to chrysin, which is converted to baicalein via F6H or to wogonin by the combined action of flavone 8-hydroxylase (F8H) and O-methyltransferase (OMT). Baicalein and wogonin then combine with sugar molecules via flavonoid 7-O-glucuronosyltransferase (UBGAT) to generate baicalin and wogonoside (Zhao et al., 2016, 2018; Zhao et al., 2019b).

The formation and accumulation of plant secondary metabolites are regulated by a complex interplay of plant hormones and transcription factors (TFs). Plant hormones, such as gibberellic acid (GA) and abscisic acid (ABA), reportedly promote flavonoid production in *S. baicalensis* (Yuan et al., 2013; He et al., 2023). Meanwhile, TFs, such as the NAC and MYB families, are key regulators of flavonoid synthesis (Yuan et al., 2013, 2015; Qian et al., 2020; Fang et al., 2023, 2023; He et al., 2023). The crosstalk between plant hormones and TFs adds another layer of regulation to secondary metabolite formation and accumulation (Bang et al., 2008; Yuan et al., 2013; Zhou et al., 2022).

RNA-seq is a robust quantitative tool for investigating gene regulation and function. In plants, this method can be applied to analyze cell and tissue transcriptional profiles and identify the key genes associated with secondary metabolite biosynthesis and their complex networks involving various hormones and TFs. In fact, Cao et al. (2022) adopted transcriptomics to demonstrate that the endophytic fungus KL27 enhances taxol accumulation in *Taxus chinensis* by regulating specific hormones and TFs to promote the expression of key genes in taxol biosynthesis. Similarly, through transcriptomic analysis, Guo et al. (2018) identified the key plant hormones and TFs involved in light-regulated anthocyanin biosynthesis in cherries.

Endophytic fungi are microorganisms that reside in the internal tissues of living plants and do not cause any immediate or overt adverse effects (Hardoim et al., 2015). Endophytic fungi have various functions in host plants, including promoting host growth and secondary metabolite accumulation (Jia et al., 2016). Previous studies have found that several material bases, such as polysaccharide, oligosaccharide, protein, and polypeptide (Li et al., 2013; Yu et al., 2016; Zhang et al., 2019; Wu et al., 2019a), from fungal mycelial extracts or culture filtrates, can promote host growth and secondary metabolite accumulation. Xu et al. (2023) used the endophytic fungus *Colletotrichum* sp. AP12 to increase the expression of key genes in the andrographolide biosynthesis pathway, promoting its synthesis and accumulation in *Andrographis paniculata*. Additionally, Ming et al. (2013) found that the endophytic fungus *Trichoderma atroviride* promotes the expression of genes related to the tanshinone biosynthesis pathway, leading to its synthesis in *Salvia miltiorrhiza*. However, the precise mechanisms through which endophytic fungi facilitate flavonoid synthesis in *S. baicalensis* remain unclear.

We previously found that treating *S. baicalensis* calli with the culture filtrate of *Fusarium solani* CL105 enhanced cell growth and flavonoid production (Zhang et al., 2023). Accordingly, the current study analyzes the effects of three elicitors from the *F. solani* CL105 culture filtrate, namely exopolysaccharide (EPS), exoprotein (EP), and other parts (OP), on the growth and flavonoid production of *S. baicalensis* calli. We then employ transcriptome analysis and quantitative real-time reverse transcription polymerase chain reaction (qRT-PCR) to explore how the active elicitor regulates flavonoid production in *S. baicalensis* calli. This study provides new insights into the mechanism by which endophytic fungi enhance flavonoid accumulation in *S. baicalensis*. Additionally, it presents new ideas for large-scale flavonoid production using biotechnological methods.

## 2 Materials and methods

### 2.1 Calli induction

*S. baicalensis* seeds, which were identified as *S. baicalensis* Georgi by Professor Yuguang Zheng of Hebei University of Chinese Medicine, were purchased from Anguo Herbal Market, Hebei Province, China. The seeds were surface sterilized according to the methods described in our previous study (Zhang et al., 2023), inoculated on a MS medium (Hopebio, Qingdao, China) (Murashige and Skoog, 1962), and cultured at 25 ± 1°C for 14 days to obtain sterile seedlings. Calli induction in *S. baicalensis* was performed as previously described, with some modifications (Wan et al., 2012). Briefly, stem explants (grown *in vitro*) were cut aseptically into approximately 10 mm segments and placed on MS medium supplemented with 2.0 mg/L 6-BA (6-Benzylaminopurine) (Xiya Reagent, Linyi, China) and 1.0 mg/L NAA (-naphthalene acetic acid) (Solarbio, Beijing, China), which could promote the induction and proliferation of calli (Xu et al., 2022). After 2 weeks, *S. baicalensis* calli were subcultured in the same culture medium at 25 ± 1°C.

### 2.2 Preparation of elicitors

The endophytic fungus *F. solani* CL105 was grown in 100 mL Potato Dextrose Broth (PDB) medium in a 250 mL Erlenmeyer flask. The fungal culture was incubated at 25 °C with shaking at 180 rpm for 7 days. After fermentation, the fermented broth was filtered, the filtrate was collected and concentrated to a third of the original volume, then mixed with four volumes of 95% ethanol and incubated at 4°C for 48 h, then filtered to obtain the supernatants and precipitates. The precipitate from the ethanol dispersion was collected as EPS, and crude protein was collected from the supernatant using the Sevage method (supernatant:liquid:chloroform:*n*-Butyl alcohol = 25:4:1, v:v:v), as EP; the crude protein elicitor was removed from the supernatant, as was OP. All the elicitors were dried using a vacuum freeze dryer (TF-FD-1, Shanghai Tianfeng Industrial Co., Ltd., Shanghai, China) and stored at 4°C.

### 2.3 Elicitor treatment

We have previously shown that 5% (v/v) culture filtrate elicitors of *F. solani* CL105 could induce flavonoid production in *S. baicalensis* calli (Zhang et al., 2023). The EPS, EP, and OP were respectively dissolved in 25 mL MS medium to prepare a series of concentrations based on their yields (760 mg/L, 192 mg/L and 1040 mg/L) in culture filtrate and the active concentrate (5%, v/v) of culture filtrate (EPS: 20, 40, 80 and 160 mg/L; EP: 5, 10 and 15 mg/L; OP: 20, 50 and 80 mg/L). MS medium without an elicitor was used as a control. The media was sterilized at 121°C for 30 min. Approximately 1.50 g of *S. baicalensis* calli were subcultured into media and incubated at 25 ± 1°C. The experiments were repeated thrice for each elicitor concentration. *S. baicalensis* calli were harvested on 0, 5, 10, and 15 days following EPS, EP, and OP treatment, and the fresh weight of *S. baicalensis* calli was measured. One part of *S. baicalensis* was frozen in liquid nitrogen and sent to Shanghai Majorbio Bio-pharm Biotechnology Co., Ltd. (Shanghai, China) for RNA sequencing. While the other part was dried at 50°C in a dry heat oven until a constant weight, and the dry weight (DW) was measured.

### 2.4 Quantification of flavonoids with HPLC

For high-performance liquid chromatography (HPLC) analysis, 0.01 g samples of *S. baicalensis* calli powder were extracted with methanol under ultrasound for 30 min. The extracts were centrifuged at 12,000 rpm for 10 min and the supernatants were filtered through a 0.22 μm organic membrane. Analyses were performed using a Shimadzu LC2030 instrument (SHIMADZU, Kyoto, Japan). A DiKMA Diamonsil C18 (4.6×250 mm, 5.0 µm) column was applied for all analyses. Baicalin, baicalein, wogonoside, and wogonin, were detected and quantified by comparison with authentic standards (Biopurify Company, Chengdu, China). The mobile phases comprised A (water containing 0.1% formic acid) and B (acetonitrile). The gradient elution program was as follows: 25% B for 0–10 min, 25–45% B for 10–30 min, and 45–55% B for 30–55 min; the detection wavelength was 254 nm. The sample injection volume was 10 μL and the column temperature was maintained at 25°C. The method was validated in terms of linearity, precision, repeatability, stability, and recovery (**Supplementary Tables 1**, **2**).

### 2.5 Transcriptome determination and analysis

#### 2.5.1 Sample preparation, RNA extraction, library preparation, and transcriptome sequencing

Total RNA was extracted from the *S. baicalensis* calli using TRIzol^®^ Reagent following the manufacturer’s instructions. RNA quality was determined using a 5300 Bioanalyzer (Agilent, Santa Clara, CA, USA) and quantified using an ND-2000 (NanoDrop Technologies, Wilmington, USA). Only high-quality RNAsample (OD260/280 = 1.8–2.2, OD260/230≥2.0, RIN≥6.5, 28S:18S≥1.0, >1 μg) was used to construct sequencing library. mRNA was enriched using oligo (dT) magnetic beads and was randomly fragmented using a fragmentation buffer. Under the action of reverse transcriptase, random hexamers were added to synthesize first-stranded cDNA using mRNA as a template. Thereafter, under DNA polymerase I, second-strand cDNA was synthesized. A poly (A) tail was added and connected to the sequence adaptors after end repair. The 300-bp cDNA target fragments were selected from the libraries on 2% low range ultra agarose gel. cDNA libraries were enriched by PCR amplification and then quantified using Qubit (*v*.4.0). A NovaSeq 6000 sequencer (2 × 150 bp read length) was used to sequence the paired-end RNA-seq (Wu et al., 2019b).

#### 2.5.2 *De novo* transcriptome assembly and gene functional annotation

Raw reads were filtered for quality using FASTQ (v.0.19.5), and the resulting clean reads were *de novo*-assembled using Trinity (v.2.8.5). To increase assembly quality, all assembled sequences were filtered using CD-Hit and translated.

All *de novo*-assembled unigenes were annotated using the following databases: NCBI non-redundant protein sequences (NR, http://www.ncbi.nlm.nih.gov); Protein family (Pfam, http://xfam.org/); Swiss-Prot (http://www.expasy.ch/sprot); Kyoto Encyclopedia of Genes and Genomes (KEGG, http://www.genome.jp) databases using Diamond to identify the proteins that had the highest sequence similarity with the given transcripts to retrieve their function annotations and a typical cut-off E-values less than 1.0×10–5 was set. The BLAST2GO program was used to obtain GO (http://www.geneontology.org) annotations of uniquely assembled transcripts to describe biological processes, molecular functions, and cellular components. Metabolic pathway analysis was performed using KEGG.

#### 2.5.3 Differentially expressed genes (DEGs) analysis

Gene expression levels were estimated using the RSEM (Li and Dewey, 2011) for each sample. Clean data were mapped onto assembled transcriptomes. The read count of each gene was obtained from the mapping results. Differential expression analysis of the two samples was performed using the DEseq2 package. Genes with *P* value < 0.05 and |log2 (fold change)| > 1 found by DEseq2 were assigned as differentially expressed.

### 2.6 Quantitative real-time PCR (qRT-PCR) validation

To validate the RNAseq gene expression, total RNA was isolated from *S. baicalensis* calli and analyzed using quantitative real-time polymerase chain reaction (qRT-PCR). Four genes, namely CHS, CHI, FNS, and F6H, associated with flavonoid biosynthesis were selected from the RNA-Seq data for qRT-PCR analysis. The constitutively expressed 18S rRNA gene was used as the housekeeping gene (Zhou et al., 2014). All specific primer pairs used in this study are listed in **Supplementary Table 3**. The 25 μL reaction mixture consisted of 1 μL Strand cDNA, 10µL qRT-PCR taq master mix (YEASEN, Shanghai, China), 0.7 µL forward primer (10 µM), 0.7 µL reverse primer (10 µM), and 7.6 µL highly pure water. The reaction was performed using an Thermofisher QuantStudio 1(Thermofisher, Waltham, MA, USA). The experiment was conducted in triplicates. The expression level of each target gene was determined using the 2-ΔΔCt method, normalized based on the CT value of the housekeeping gene.

### 2.7 Statistical analysis

The fresh and dry weight, flavonoids contents, and relative-expression level of *S. baicalensis* calli were expressed as mean ± SD from three separate observations. Data were analyzed using one-way analysis of variance (ANOVA) and least significant difference (LSD) using SPSS19.0 software. p value < 0.05 was considered statistically significant.

## 3 Results

### 3.1 Effects of elicitors on *S. baicalensis* calli growth

**Figure 1:**
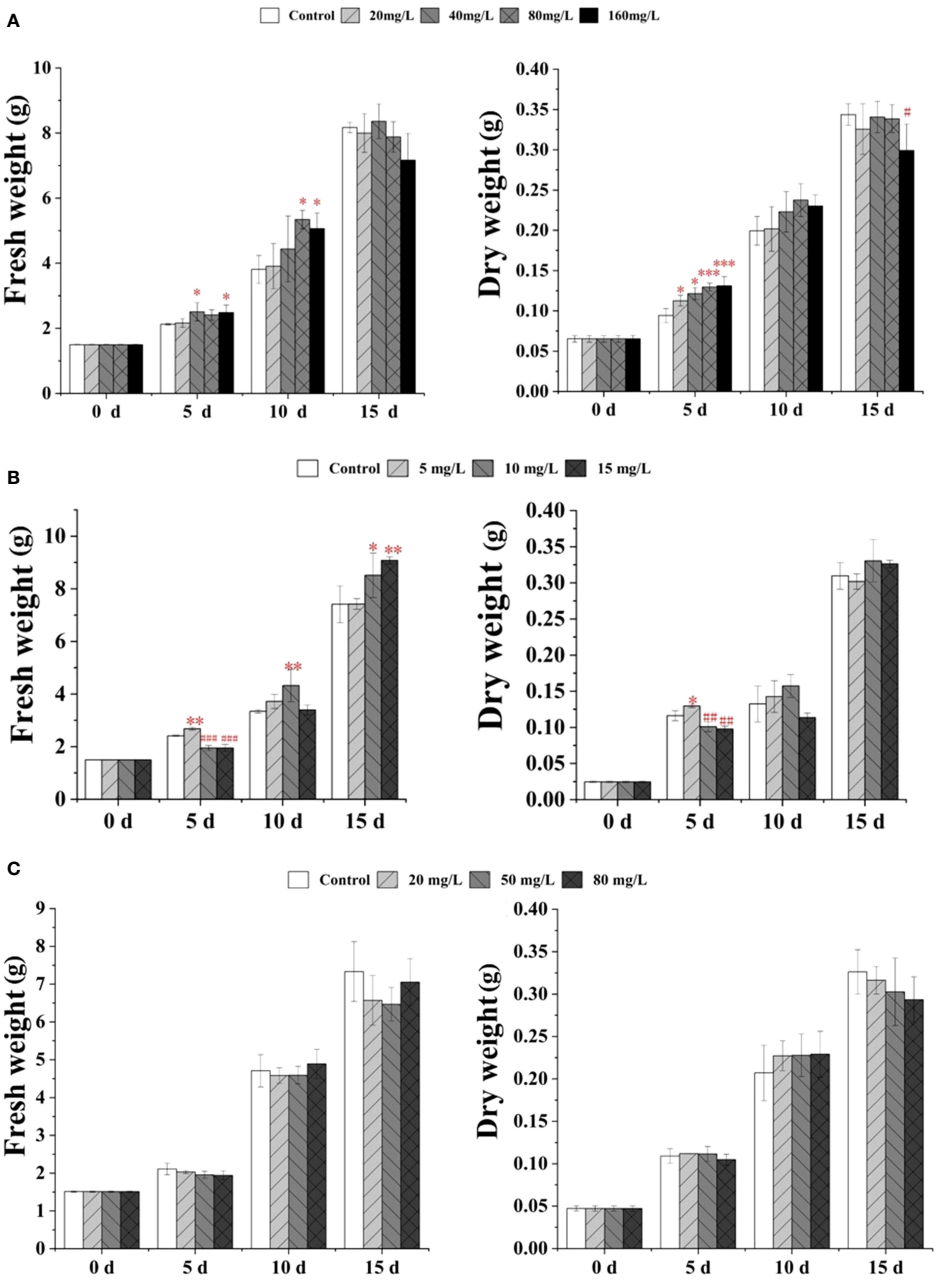
Effect of EPS **(A)**, EP **(B)** and OP **(C)** on growth in *S. baicalensis* calli. Data are presented as means ± SD, n = 3. *P<0.05, **P<0.01, ***P<0.001; #P<0.05, ##P<0.01, ###P<0.001.

As shown in **Figure 1A**, with 40 or 160 mg/L EPS treatments for 5 days, the fresh weight of *S. baicalensis* calli was significantly increased by ~1.18- and 1.17-fold compared with the control. Meanwhile, after treatment with 20, 40, 80, or 160 mg/L EPS for 5 days, the dry weight was significantly increased by ~1.20- to 1.39-fold compared with the control. After 10 days of culture, the fresh weight of *S. baicalensis* calli was significantly increased following treatment with 80 or 160 mg/L EPS by ~1.19- and 1.15-fold, respectively. However, reductions were observed in the fresh and dry weights at 15 days after culture. In particular, the dry weight of *S. baicalensis* calli was significantly reduced with 160 mg/L EPS treatment (~13% decrease) compared with the control.

The fresh weight of *S. baicalensis* calli continuously increased following EP treatment from 5 to 15 days, while the dry weight was significantly increased only after 5 days of treatment with 5 mg/L EP (1.12-fold; **Figure 1B**). In contrast, the fresh and dry weights were significantly decreased following treatment with 10 mg/L (18% and 13%) or 15 mg/L (19% and 15%) EP for 5 days compared with the control. After 10 days of culture with 10 mg/L EP, the fresh weight of *S. baicalensis* calli significantly increased by ~1.30-fold compared with the control. After 15 days of treatment with 10 mg/L or 15 mg/L EP, the fresh weight significantly increased by ~1.15- and 1.22-fold, respectively, compared with the control.

Treatment with OP at 20, 50, or 80 mg/L did not significantly impact on the growth of *S. baicalensis* calli after 5, 10 or 15 days (**Figure 1C**).

### 3.2 Effects of elicitors on *S. baicalensis* calli flavonoid production

**Figure 2:**
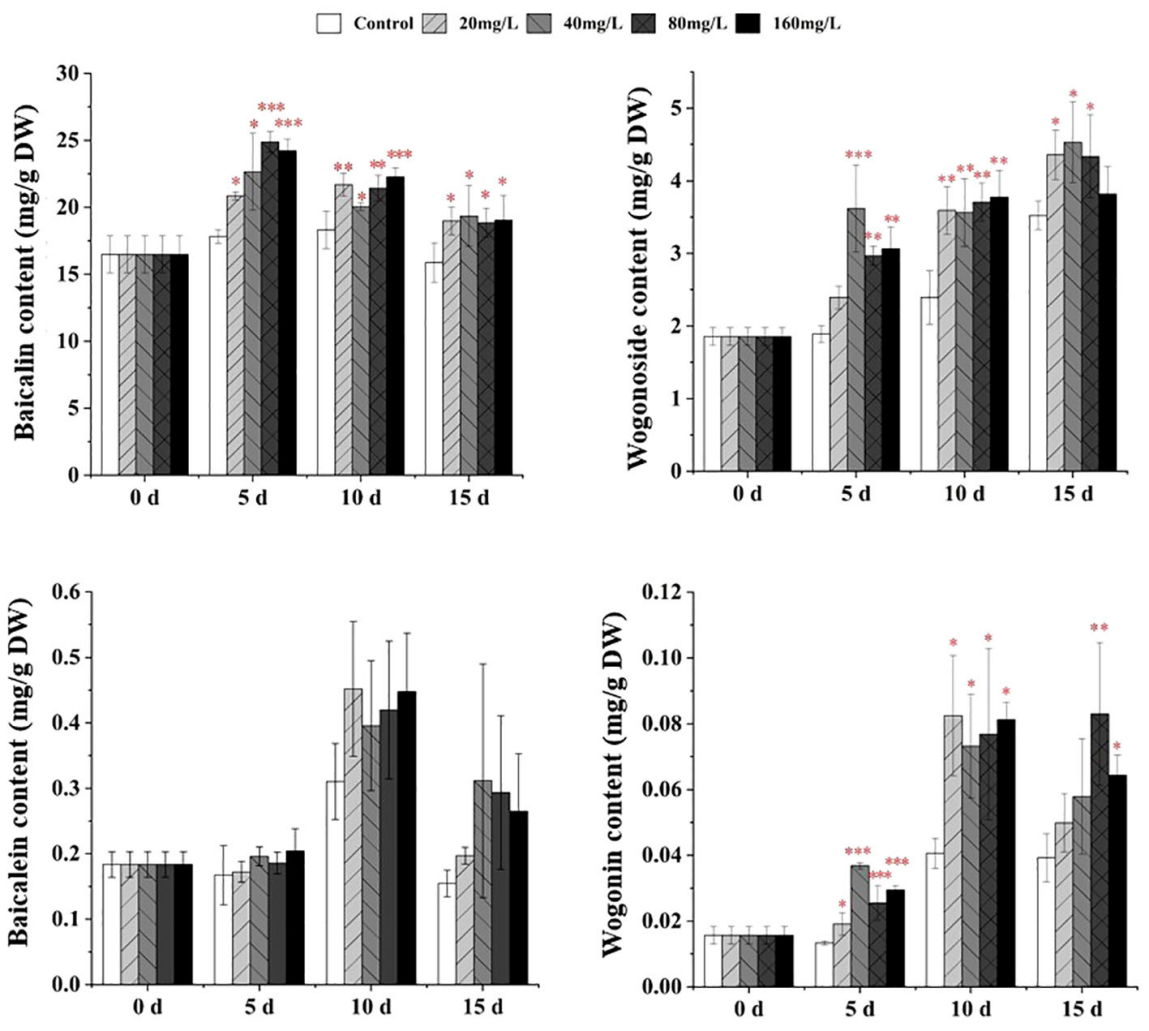
Effects of EPS on the accumulation of flavonoids in *S. baicalensis* calli on days 5, 10, and 15, respectively. The treatments were control, 20, 40, 80, and 160 mg/L. Data are presented as means ± SD, n = 3. *P<0.05, **P<0.01, ***P<0.001.

The contents of baicalin, wogonoside, and wogonin in *S. baicalensis* calli were significantly increased following EPS treatment for 5–15 days (**Figure 2**). After 5 days of culture with 20, 40, 80, or 160 mg/L EPS, baicalin and wogonin levels were significantly increased by ~1.17–2.76-fold. Meanwhile, the wogonoside content was significantly increased following treatment with 40, 80, or 160 mg/L EPS by ~1.51–1.91-fold compared with the control. After culturing for 10 days with any concentration of EPS, the contents of baicalin, wogonoside, and wogonin were significantly increased by ~1.10 to 2.03-fold compared with the control. Similarly, after 15 days, the baicalin content was significantly increased by ~1.19–1.22-fold following treatment with all EPS concentrations. However, after 15 days, the wogonoside content was only significantly increased following treatment with 20, 40, or 80 mg/L EPS (1.23–1.28-fold) and the wogonin content was significantly increased following 80 mg/L (2.11-fold) or 160 mg/L (1.64-fold) EPS treatment.

EP had a weak effect on flavonoids. After culturing for 5 days with 5 mg/L EP, the baicalin content was significantly increased by ~1.06-fold compared with the control (**Supplementary Figure 1**). At the late stage of induction, OP significantly promoted flavonoid synthesis. After culturing for 15 days with 50 or 80 mg/L OP, the wogonoside contents were significantly increased by ~1.45- and 1.31-fold, respectively, while those of baicalein and wogonin were significantly increased following 80 mg/L OP treatment by ~2.48-fold and 1.61-fold, respectively, compared with the control (**Supplementary Figure 2**).

### 3.3 Transcriptomic analysis and differentially expressed genes

Since EPS is the main active elicitor, we performed transcriptome sequencing on EPS-treated *S. baicalensis* calli. In total, 212.11 Gb of clean data were generated from the samples. All clean reads were subjected to *de novo* assembly using Trinity, producing 94,597 transcripts and 94,597 unigenes. Sequencing data quality evaluations are listed in **Supplementary Table 4**. The unigenes were functionally annotated based on the seven largest public databases. A total of 38,106 unigenes (40.28% of the total unigenes) were annotated; 19,209 unigenes (50.41% of the total annotated unigenes) were matched in the Gene Ontology (GO) database, and 9,942 (26.09%), 19,740 (51.80%), 22,154 (58.14%), 17,482 (45.88%), and 22,667 (45.27%) unigenes exhibited significant similarity to sequences in the GO, KEGG, eggNOG, NCBI non-redundant protein sequences (NR), Swiss-Prot, and Pfam databases, respectively.

All unigenes were searched against the GO database to classify their functions based on the NR annotation. The 19,209 unigenes assigned to one or more GO terms were classified into three main GO categories and 53 groups (**Supplementary Figure 3**). Within the “biological process” domain, the most evident matches were “cellular process” (8,198), “metabolic process” (7,239), and “biological regulation” (3,074). In the “cellular component” domain, “cell part” (9,774), “membrane part” (7,431), and “organelle” (5,641) predominated. For the “molecular function” domain, the genes were primarily enriched in “binding” (10,479) and “catalytic activity” (10,123).

For further analysis, the unigenes were mapped onto the KEGG database to categorize gene function and identify biochemical pathways. A total of 9,942 unigenes were annotated and assigned to five main KEGG metabolic pathways, 18 sub-branches, and 5,814 KEGG pathways. The most common sub-branch was “translation” (954), followed by “carbohydrate metabolism” (862) and “folding, sorting, and degradation” (590) (**Supplementary Figure 4**). An additional 147 unigenes matched “phenylpropanoid biosynthesis” (ko00940) and 54 unigenes matched “flavonoid biosynthesis” (ko00941).

**Figure 3:**
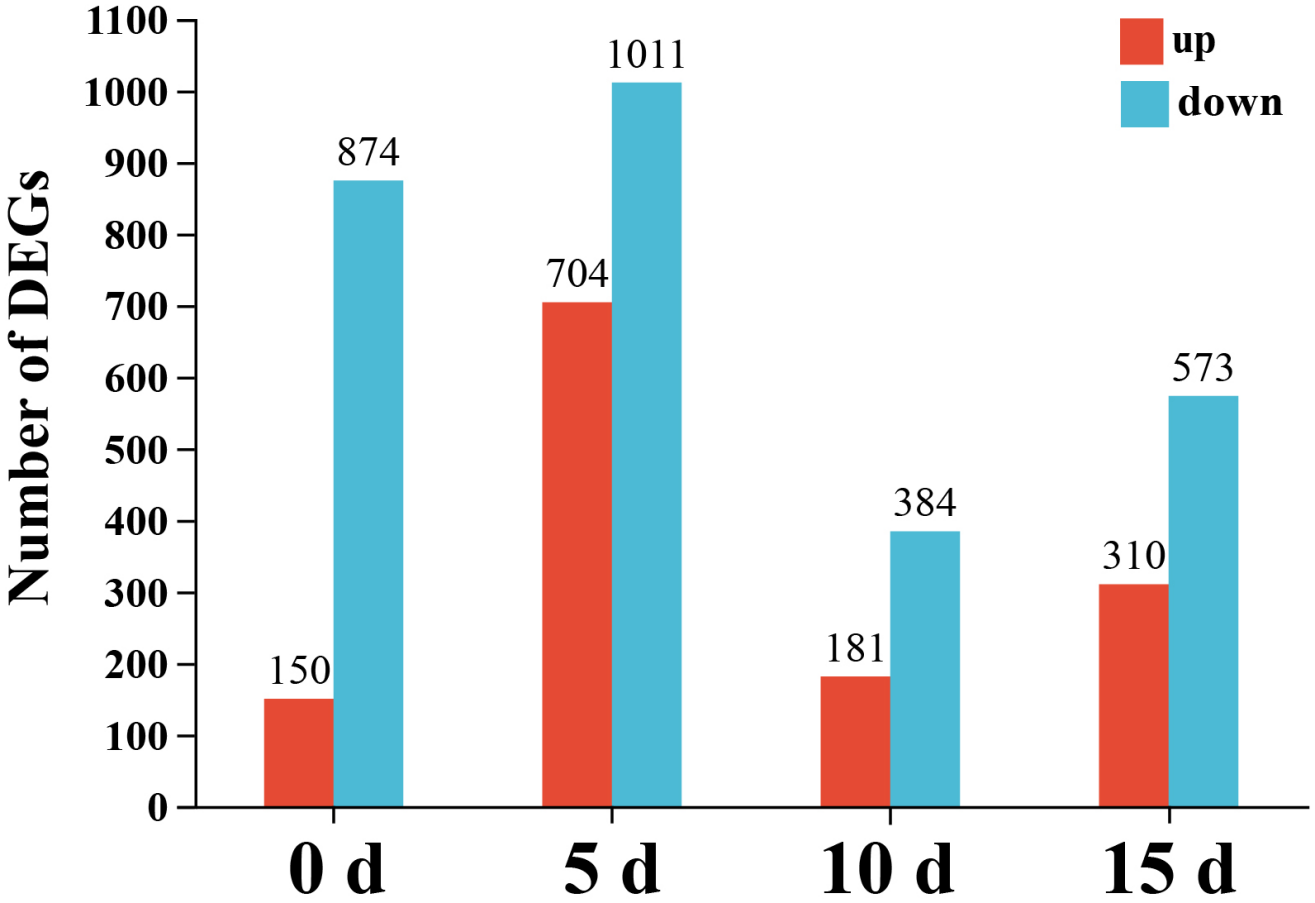
Number of DEGs at four comparisons. The abscissa represents EPS-treated versus untreated at different time points (0, 5, 10 and 15 days), and the ordinate represents the number of the up- and down-regulated DEGs.

A gene was designated as differentially expressed based on the following cutoff criteria: |Log2FC| ≥ 1 and P < 0.05. A total of 1,024, 1,715, 565, and 883 DEGs were identified in the EPS-treated for 0 d vs. control, EPS-treated for 5 days vs. control, EPS-treated for 10 days vs. control, and EPS-treated for 15 days vs. control comparisons, respectively. Of these DEGs, 150, 704, 181, and 310 were up-regulated, whereas 874, 1,011, 384, and 573 were down-regulated at 0, 5, 10, and 15 days, respectively (**Figure 3**).

A volcano plot was constructed to illustrate the distribution of the DEGs among the four comparison groups. The number of DEGs in the 5-day group was higher than in the 10- or 15-day groups. Moreover, the expression levels of a few genes in the 5-day group were significantly up-regulated with a higher degree of significance compared with the 10- or 15-day groups (**Supplementary Figure 5**).

### 3.4 DEGs involved in flavonoid biosynthesis and qRT-PCR validation

**Figure 4:**
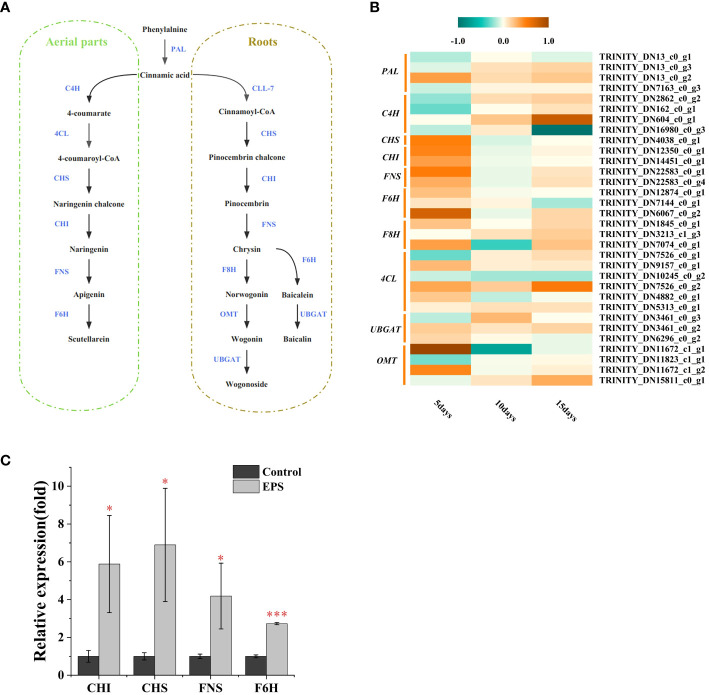
DEGs involved flavonoid biosynthesis and qRT-PCR validation. **(A)** Biosynthetic pathways of the flavonoids in *S. baicalensis.* Enzymes abbreviations are: PAL: phenylalanine ammonialyase; C4H: cinnamate 4-hydroxylase; 4CL: 4-coumarate CoA ligase; CHS: chalcone synthase; CHI: chalcone isomerase; FNS: flavone synthase; F6H: flavone 6-hydroxylase; CLL-7: cinnamate-CoA ligase; F8H: flavone 8-hydroxylase; OMT: O-methyltransferase; UBGAT: flavonoid 7-O-glucuronosyltransferase. **(B)** Expression analysis of the flavonoid biosyntheisis-related unigenes at 5, 10 and 15days. The bar indicated the “log2 (fold change)”. **(C)** Effects of EPS on the expression of flavonoid biosynthesis key genes in *S. baicalensis* calli. Data are presented as means ± SD, n = 3. *P<0.05, ***P<0.001.

The flavonoid biosynthesis pathway in *S. baicalensis* has been previously described (Hu et al., 2022; Pei et al., 2022). To further analyze how the identified DEGs contribute to higher flavonoid accumulation following EPS treatment, their expression patterns in flavonoid biosynthesis pathways were analyzed (**Figure 4A**). As shown in **Figure 4B**, most genes involved in flavonoid biosynthesis were up-regulated in *S. baicalensis* calli following treatment with EPS after 5 days compared with the control. Particularly evident was the expression of genes encoding CHS, CHI, FNS, and F6H. However, ≤ 50% were up-regulated at 10 or 15 days. Hence, EPS promoted flavonoid accumulation by up-regulating genes encoding enzymes in the flavonoid biosynthesis pathway. Activation of the flavonoid biosynthesis pathway by EPS occurs in the early stages of stimulation and decreases over time.

To assess the reliability of our transcriptomic data, four up-regulated genes associated with the flavonoid biosynthesis pathway in *S. baicalensis* calli during 5-day cultures were selected for validation via RNA-seq. The expression profiles for the four DEGs determined via qRT-PCR were consistent with those obtained via transcriptomics (**Figure 4C**, **Supplementary Table 5**). Therefore, these findings suggest that the transcriptome information is reliable.

### 3.5 DEGs involved in hormone biosynthesis

Phytohormones influence the expression of key enzymes in the flavonoid biosynthesis pathway (Yu et al., 2021). Our transcriptome data showed that the expression of genes encoding enzymes in GA, ABA, ethylene (ET), and brassinosteroid (BR) biosynthesis was significantly increased. The diterpenoid biosynthesis, carotenoid biosynthesis, cysteine and methionine metabolism, and brassinosteroid biosynthesis pathways occur in response to GA, ABA, ET, and BR biosynthesis. After EPS treatment for 5 days, the genes encoding GA2ox (K04125) in GA biosynthesis, CYP707A (K09843) in ABA biosynthesis, ERF1 (K14516) in ET biosynthesis, and CYP85A1 (K09590) and CYP92A6 (K20623) in BR biosynthesis were significantly differentially expressed compared to the control. *GA2ox*, *CYP707A*, and *CYP85A1* were significantly up-regulated, while, *CYP92A6* and *ERF1* were down-regulated (**Supplementary Table 6**).

### 3.6 DEGs involved in transcription factors expression

A total of 857 unigenes were annotated to TFs, belonging to 34 families (**Supplementary Table 7**). The top TF families included the MYB_superfamily (143 unigenes), AP2/ERF (93 unigenes), C2C2 (68 unigenes), bHLH (59 unigenes), WRKY (59 unigenes), NAC (56 unigenes), and GRAS (44 unigenes). Following EPS treatment for 5 days, 122 DEGs were significantly up-regulated and 106 down-regulated. After EPS treatment for 10 days, 24 DEGs were significantly up-regulated and 30 down-regulated. After EPS treatment for 15 days, 60 DEGs were significantly up-regulated and 55 down-regulated. Hence, the most TFs were significantly up-regulated after 5 days of EPS treatment; those related to flavonoid synthesis were primarily concentrated in the MYB superfamily (Yuan et al., 2015; Qian et al., 2020; Fang et al., 2023); however, they only included MYB3, MYB8, and MYB13.

## 4 Discussion

The stimulatory effects of EPS and EP on the growth of *S. baicalensis* calli were observed. The fresh and dry weights of *S. baicalensis* calli were significantly increased following EPS treatment, consistent with previous findings (Li et al., 2013; Zhong et al., 2016). Moreover, the fresh and dry weights of *S. baicalensis* calli were promoted within the early and middle stages of treatment, i.e., 5–10 days, consistent with the results of Zhong et al. (2016). We observed similar results for EP treatment, whereas Xu et al. (2009) reported that the EP from *Phytophthora boehmeriae* culture filtrate had no effect on host growth. We speculate that the EP from endophytic fungi has different physiological effects due to differences in structures.

In our study, EPS and OP elicited stimulatory effects on *S. baicalensis* calli flavonoid production. Different concentrations promoted baicalin, baicalein, and wogonin biosynthesis across all time points. This strong effect elicited by EPS is consistent with previous findings (Li et al., 2013; Zhong et al., 2016; Chen et al., 2022). The maximum stimulatory effects on flavonoid production in *S. baicalensis* calli were observed after 5 days of EPS treatment. Zhong et al. (2016) showed that the highest rutin and quercetin contents in tartary buckwheat sprout cultures were obtained 9 days following EPS treatment, whereas the rutin and quercetin contents showed a decreasing trend at 10 days. We speculate that EPS elicits chemical defense responses in *S. baicalensis* calli and rapidly stimulates flavonoid production following exposure. With prolonged exposure, flavonoids may break down into compounds that are harmless to the host. In addition, the high concentration of OP showed mild promotional effects on baicalein, wogonoside, and wogonin production during the late stage of culture. However, the main components of OP remain unknown. Data on the effects of other elicitors (beyond exopolysaccharides and exoproteins) from the culture filtrates of endophytic fungi on the synthesis of plant secondary metabolites are lacking.

Interestingly, our results indicated that EPS exhibited a slight growth-promoting effect and, promoted flavonoid synthesis in *S. baicalensis* calli. Similar findings have been observed in previous studies. The polysaccharide derived from *Pestalotiopsis* sp. DO14 not only enhanced the accumulation of flavonoids in *Dendrobium ferrugineum* seedlings, but also showed a slight improvement in the growth (1.19–1.35-fold, fresh weight) (Zhu et al., 2018). Ming et al. (2013) found that polysaccharides from *T. atroviride* D16 not only promoted the growth of *S. miltiorrhiza* hairy roots, but also markedly promoted the synthesis of tanshinones (5–66-fold).

Transcriptome analysis was adopted to investigate the mechanisms underlying the cell growth and flavonoid production in *S. baicalensis* calli treated with 80 mg/L EPS and cultured for 0, 5, 10, and 15 days. The number of DEGs initially increases and then decreases over time. We speculate that EPS rapidly stimulates the expression of DEGs in the early stage of treatment. With prolonged exposure, the number of DEGs decreases due to attenuation of the stimulation. CHS, CHI, FNS, and F6H were identified as being involved in the early stages of EPS induction. qRT-PCR analysis confirmed that after culturing for 5 days, the transcription levels of *CHS*, *CHI*, *FNS*, and *F6H* were higher in EPS-treated *S. baicalens*is calli than in the control. This suggests that the promotion of flavonoid biosynthesis by EPS occurred primarily during the initial stages of induction. Moreover, the increased accumulation of baicalin, wogonoside, baicalein, and wogonin in EPS-treated *S. baicalensis* calli correlated with the increased expression of the above genes. Although no biotic elicitors of *S. baicalensi* have been reported, abiotic elicitors promote flavonoid accumulation in *S. baicalensis* by up-regulating key genes in the flavonoid biosynthesis pathway. Park et al. (2011) reported that following treatment of *S. baicalensis* cell suspensions with methyl jasmonate, the expression of *CHI* increased. Meanwhile, Xu et al. (2010) found that the expressions of *PAL1*, *PAL2*, *PAL3*, *C4H*, *4CL*, and *CHS* in *S. baicalensis* cell suspensions were increased following methyl jasmonate induction. Hence, we speculate that different types of elicitors stimulate key gene expression in the flavonoid biosynthesis pathway in *S. baicalensis.*

ABA and GA can promote flavonoid synthesis in *S. baicalensis* (Yuan et al., 2013; He et al., 2023). Our RNA-seq results revealed significant up-regulation of *GA2ox* and *CYP707A*, which are involved in GA and ABA synthesis, respectively. Therefore, we speculate that EPS might promote the synthesis of GA and ABA by promoting the expression of *GA2ox* and *CYP707A*, ultimately promoting flavonoid synthesis. Although we also found that ET and BR were differentially expressed in this culture, it remains unclear whether they participate in the flavonoid synthesis process in *S. baicalensis*.

**Figure 5:**
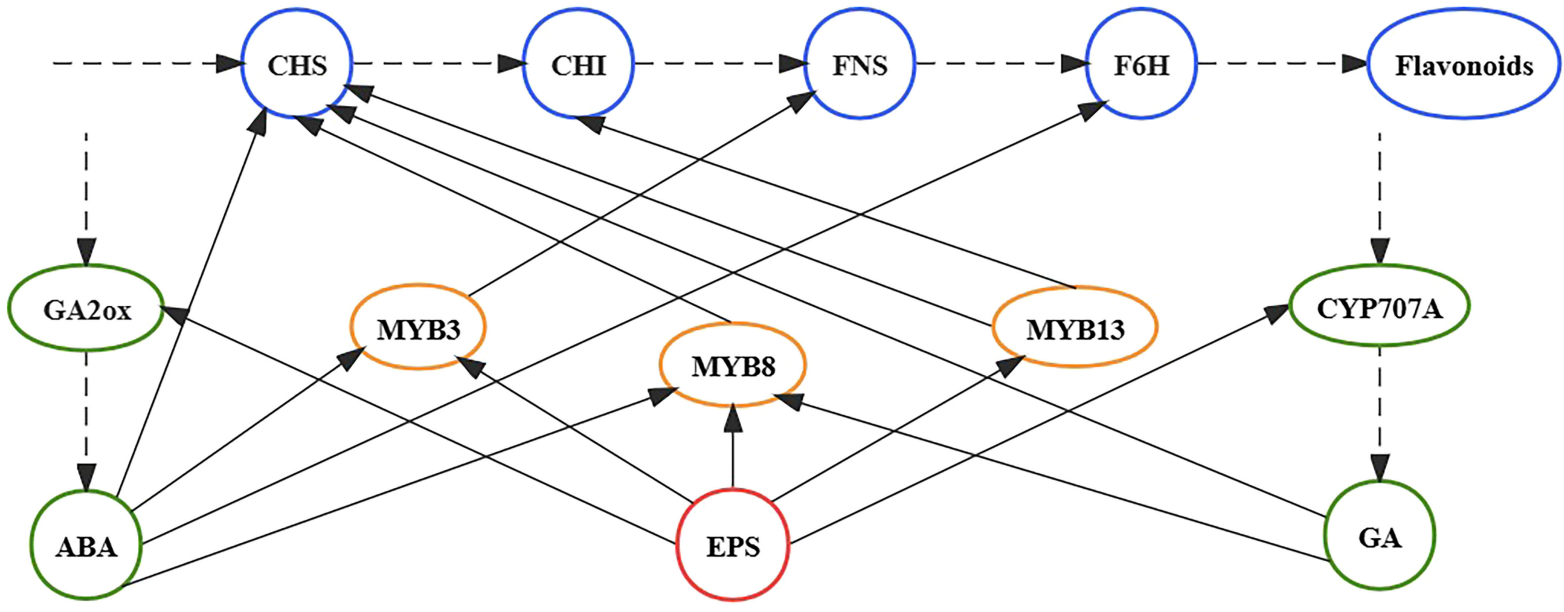
Schematic diagram of the EPS regulation of the flavonoid biosynthesis pathway in *S. baicalensis* calli. The solid arrow indicates a promoting effect. The dotted arrow indicates the biosynthetic pathways.

TFs play important roles in the growth and development of plants as they adapt to changes in the external environment (Strader et al., 2022). TFs with regulatory roles in the flavonoid synthesis pathway are concentrated in the NAC and MYB families (Yuan et al., 2013, 2015; Qian et al., 2020; Fang et al., 2023, 2023; He et al., 2023). After 5 days of EPS treatment, *MYB3*, *MYB8*, *MYB13*, *FNS*, *CHS*, and *CHI* expression was markedly up-regulated compared with the control. Hence, we posit that EPS might promote the expression of enzyme-encoding genes (*CHS*, *CHI*, and *FNS*) by up-regulating the expression of TFs (*MYB3*, *MYB8*, and *MYB13*), effectively promoting flavonoid synthesis (**Figure 5**). Interestingly, studies have shown that phytohormones can also promote the expression of TFs. For example, Yuan et al. (2013) showed that GA metabolism contributes to flavonoid biosynthesis, with the expression of *MYB8* significantly improving in *S. baicalensis* within 3 h of GA treatment. Furthermore, ABA can improve the expression of *MYB3* and *MYB8* in a relatively short period of time (Bang et al., 2008; Zhou et al., 2022). Accordingly, EPS not only stimulates the expression of ABA and GA key enzyme genes (*GA2ox* and *CYP707A*) in *S. baicalensis* calli but also promotes the expression of *MYB3*, *MYB8*, and *MYB13*. Based on our experimental results and relevant literature (Bang et al., 2008; Yuan et al., 2013, 2015; Qian et al., 2020; Zhou et al., 2022; Fang et al., 2023; He et al., 2023), EPS treatment induces flavonoid synthesis in *S. baicalensis* calli by regulating the expression of phytohormones, TFs, and enzymes. That is, EPS might stimulate the expression of genes encoding MYB3, MYB8, MYB13, GA2ox, and CYP707A, leading to significantly higher expression of *CHS*, *CHI*, *FNS*, and *F6H* and ultimately promoting the synthesis of flavonoids.

This study has certain limitations. First, although our transcriptomic analysis provided a large amount of data, we focused solely on genes related to flavonoid biosynthesis pathways. Thus, further analysis is required to identify other genes associated with the effect elicited by EPS on *S. baicalensis* calli. Second, we observed that EPS also promoted *S. baicalensis* calli growth. However, EPS is a crude polysaccharide and the active components are still unknown. It is, therefore, necessary to research the mechanisms promoting growth and the structural characterization of EPS in future studies.

## 5 Conclusion

In this study, EPS from the culture filtrate of the endophytic fungus *F. solani* CL105 constituted the main active elicitor involved in the promotion of growth and flavonoid production in *S. baicalensis* calli. Transcriptome and qRT-PCR analyses further revealed that CHS, CHI, FNS, and F6H participate in regulating the flavonoid biosynthesis pathways following EPS treatment for 5 days. Moreover, the genes encoding GA2ox and CYP707A were significantly up-regulated and associated with GA and ABA biosynthesis, respectively. Meanwhile, the expression levels of TFs, including *MYB3*, *MYB8*, and *MYB13*, were significantly up-regulated. We speculate that EPS stimulates the expression of genes encoding MYB3, MYB8, MYB13, GA2ox, and CYP707A, which leads to significantly increased *CHS*, *CHI*, *FNS*, and *F6H* expression levels and promotion of baicalin, wogonoside, baicalein, and wogonin accumulation in *S. baicalensis* calli. Our findings revealed the mechanism by which *F. solani* CL105 promotes flavonoid accumulation in *S. baicalensis* calli. This study contributes to the growing body of evidence supporting the role of endophytic fungi in regulating the accumulation of active compounds in *S. baicalensis* and provides insights for the large-scale synthesis of flavonoids through biotechnological methods. However, the mechanisms promoting growth and the structural characterization of EPS require further investigation.

## Data availability statement

The datasets presented in this study can be found in online repositories. The names of the repository/repositories and accession number(s) can be found below: BioProject, PRJNA999053.

## Author contributions

XC: Conceptualization, Data curation, Investigation, Methodology, Writing – original draft, Writing – review & editing. XZ: Conceptualization, Data curation, Investigation, Methodology, Writing – original draft, Writing – review & editing. HS: Investigation, Methodology, Writing – review & editing. YZ: Funding acquisition, Project administration, Supervision, Writing – review & editing. CS: Conceptualization, Funding acquisition, Project administration, Investigation, Supervision, Writing – review & editing.
