# Supplementary material for: Effects of elicitors from culture filtrate of Fusarium solani CL105 on flavonoid production of Scutellaria baicalensis calli
Source: Front Plant Sci. 2024 Jun 4;15:1383918. doi: 10.3389/fpls.2024.1383918 (PMC11186380; doi:10.3389/fpls.2024.1383918)
Supplement: Supplementary file 1 [file DataSheet_1.docx]

Supplementary Material

**Supplementary Figures**


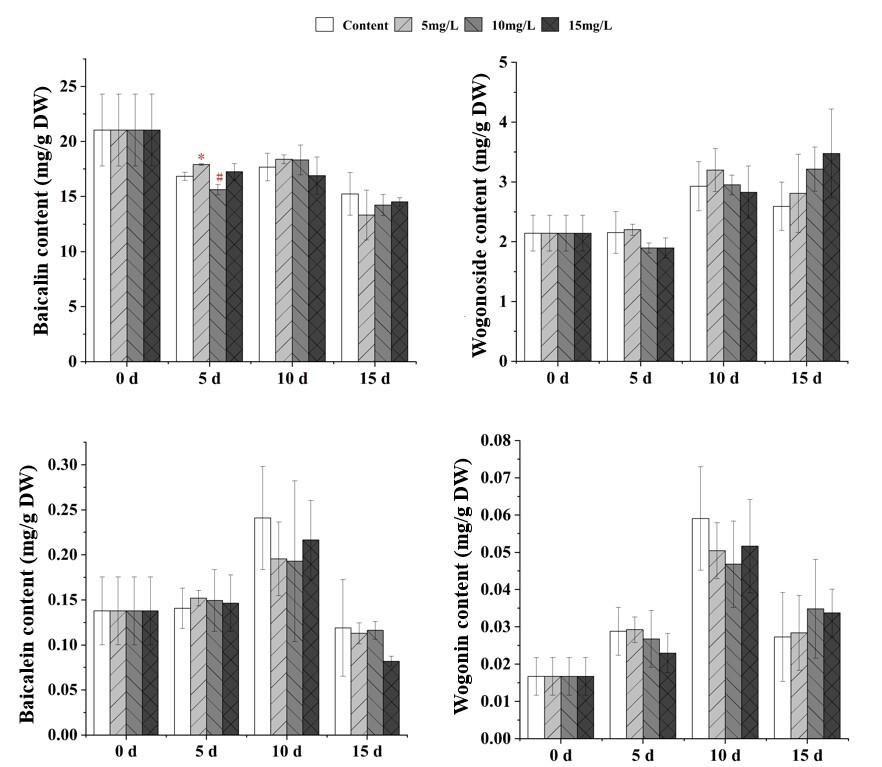


**Supplementary Figure 1.** Effects of EP on the accumulation of flavonoids in *S. baicalensis* calli on days 5, 10, and 15, respectively. The treatments were control, 5, 10, and 15 mg/L. Data are presented as means ± SD, n = 3. *P<0.05; #P<0.05.


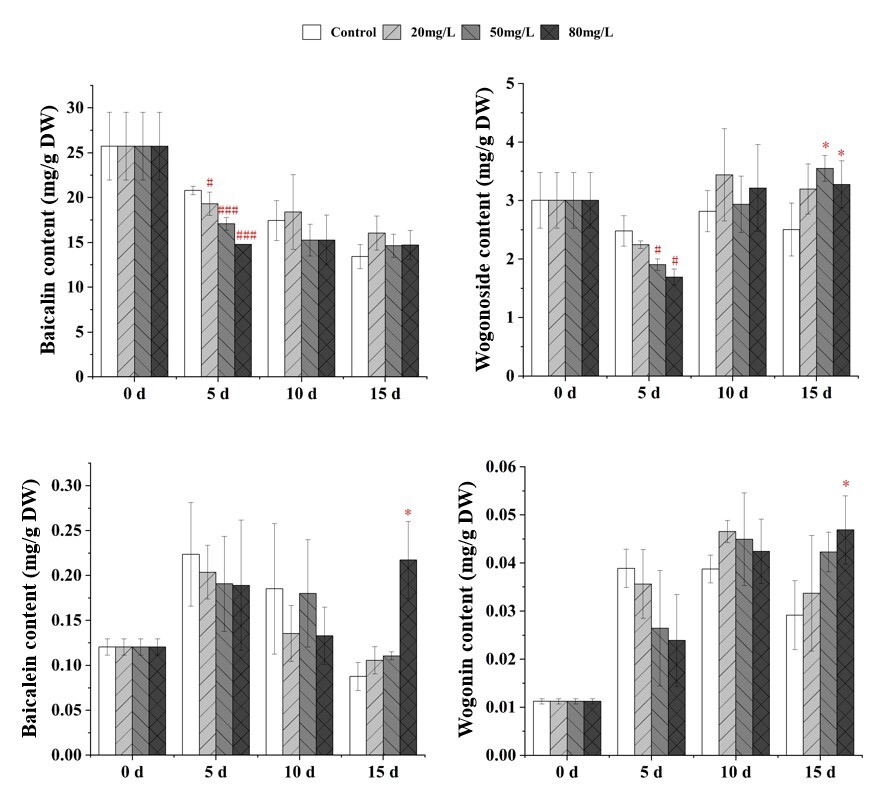


**Supplementary Figure 2.** Effects of OP on the accumulation of flavonoids in *S. baicalensis* calli on days 5, 10, and 15, respectively. The treatments were control, 20, 50, 80 mg/L. Data are presented as means ± SD, n = 3. *P<0.05; #P<0.05, ###P<0.001.


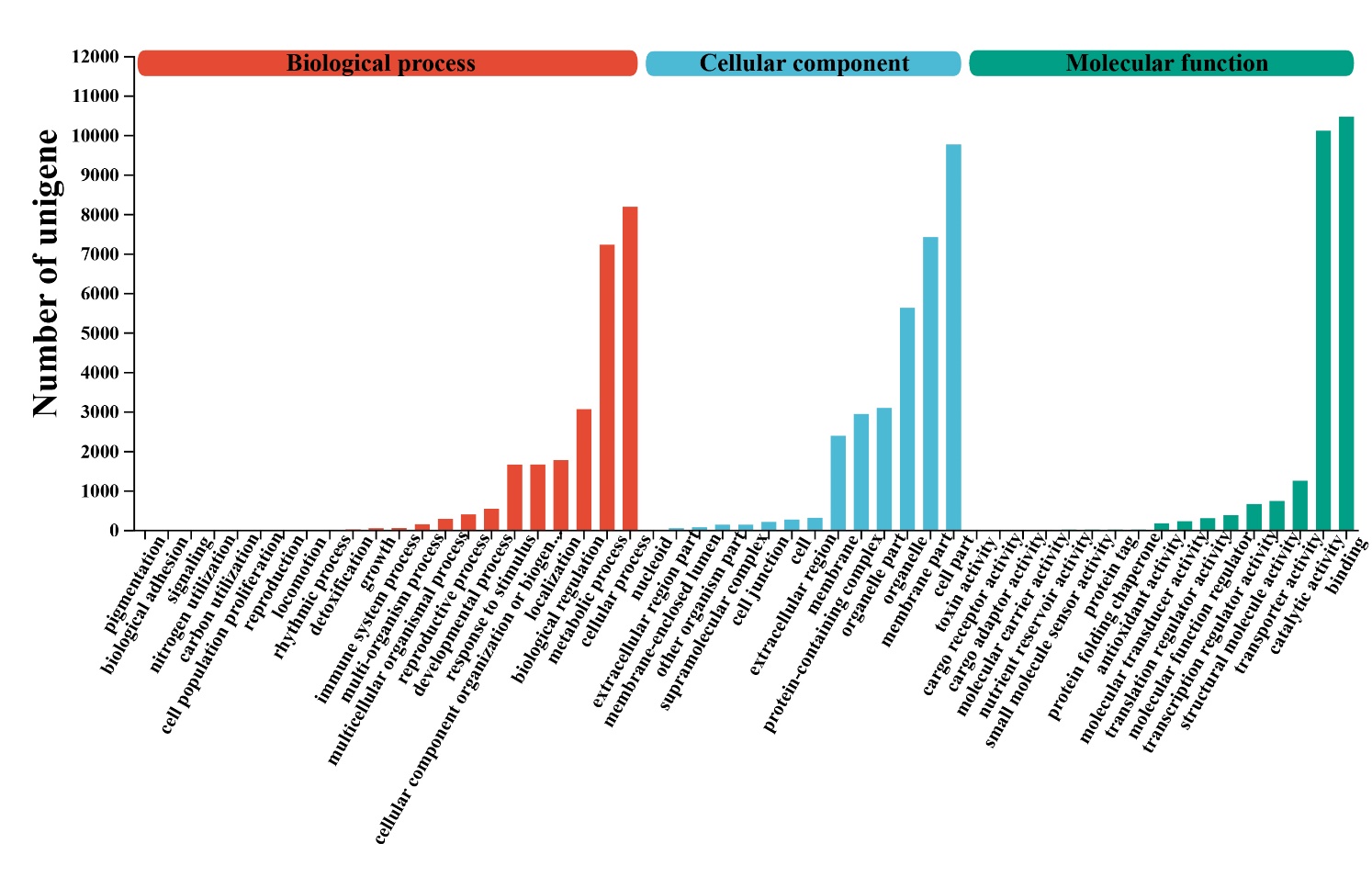


**Supplementary Figure 3.** Gene Ontology (GO) functional classification of assembled unigenes. A total of 22 groups in the “biological process” domain, 14 in the “cellular component” domain, and 17 in the “molecular function” domain.


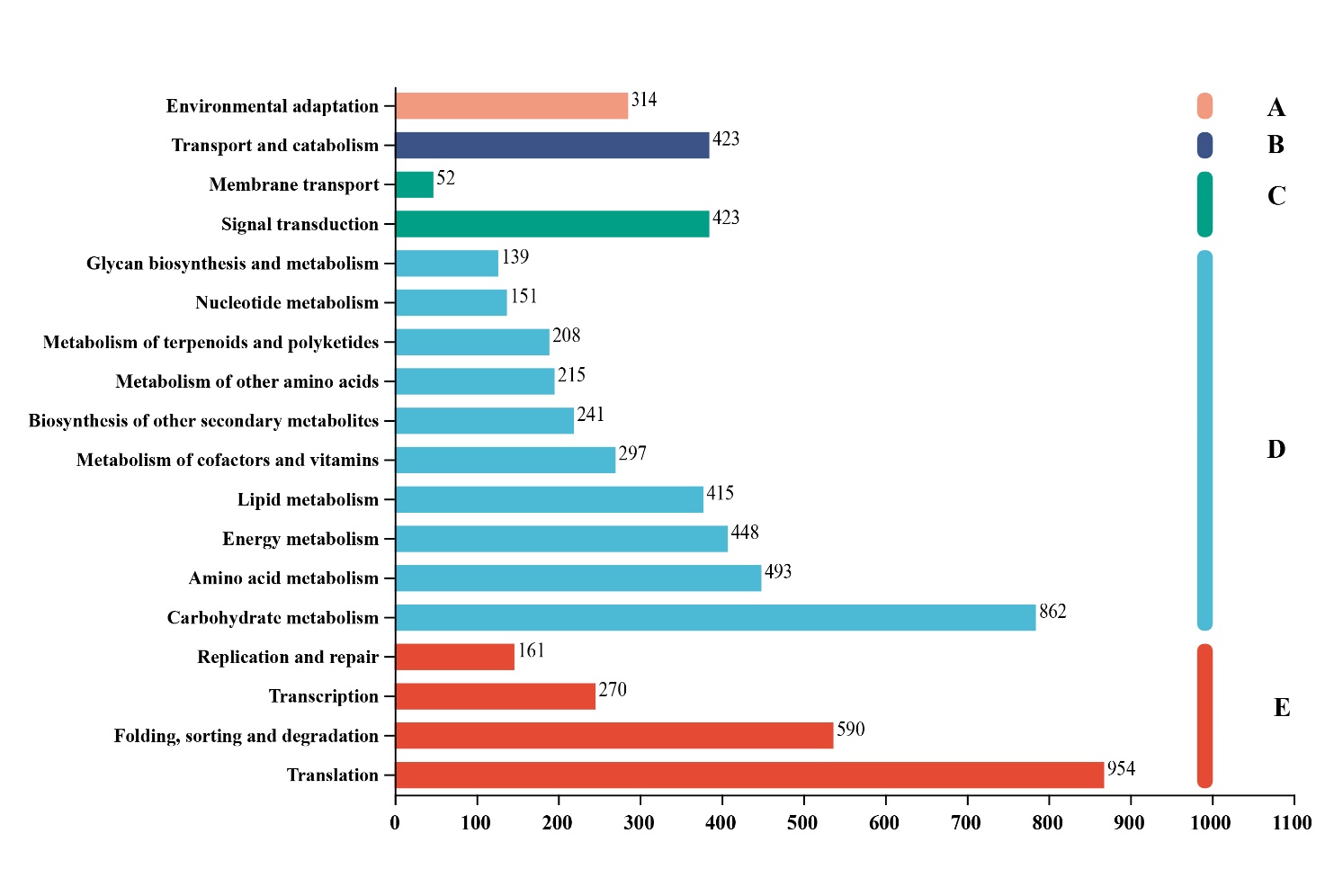


**Supplementary Figure 4.** Functional classification and pathway assignment of assembled unigenes using Kyoto Encyclopedia of Genes and Genomes (KEGG). Genetic information processing (A), metabolism (B), environmental information processing (C), cellular processes (D), and organismal systems (E). The y-axis indicates the name of the KEGG metabolic pathway, while the x-axis indicates the number of unigenes annotated to the KEGG metabolic pathway and the ratio of their number to the total number of annotated unigenes.


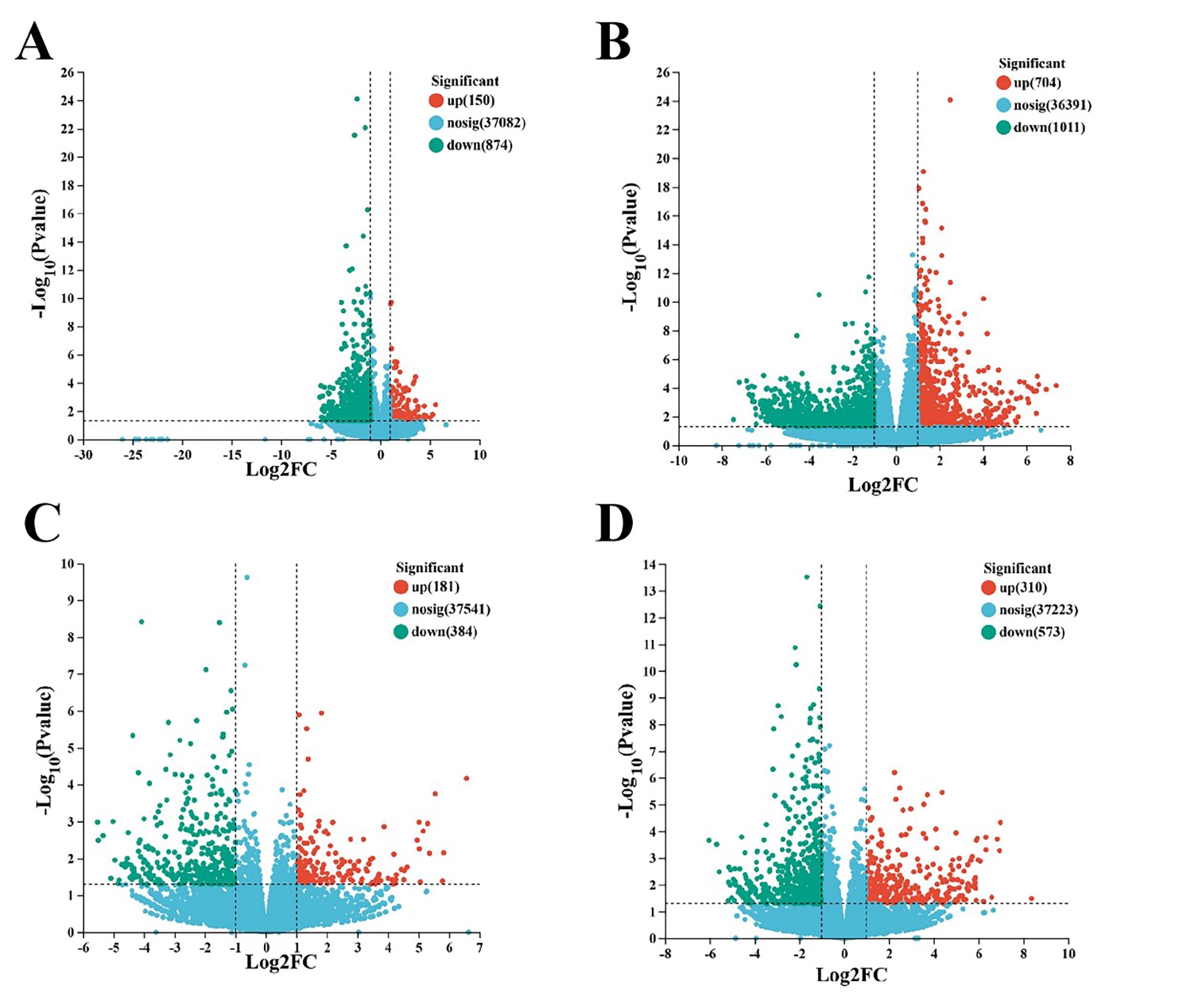


**Supplementary Figure 5.** Volcano plot illustrating the distribution of DEGs in the four comparison groups. (A) EPS-treated *S. baicalensis* calli cultured for 0 d vs. *S. baicalensis* calli cultured 0 days. (B) EPS-treated *S. baicalensis* calli cultured for 5 days vs. *S. baicalensis* calli cultured for 5 days. (C) EPS-treated *S. baicalensis* calli cultured for 10 days vs. *S. baicalensis* calli cultured for 10 days. (D) EPS-treated *S. baicalensis* calli cultured for 15 days vs. *S. baicalensis* calli cultured for 15 days.
